# Prevalence of Multimorbidity of Chronic Noncommunicable Diseases in Brazil: Population-Based Study

**DOI:** 10.2196/29693

**Published:** 2021-11-25

**Authors:** Xin Shi, Simone Maria da Silva Lima, Caroline Maria de Miranda Mota, Ying Lu, Randall S Stafford, Corintho Viana Pereira

**Affiliations:** 1 School of Maths and Information Science Shandong Technology and Business University Yantai China; 2 Business School Manchester Metropolitan University Manchester United Kingdom; 3 Management Engineering Department Universidade Federal de Pernambuco Recife Brazil; 4 Department of Biomedical Data Science School of Medicine Stanford University Stanford, CA United States; 5 Department of Medicine School of Medicine Stanford University Stanford, CA United States; 6 Otoface Recife Recife Brazil

**Keywords:** multimorbidity, prevalence, health care, public health, Brazil, logistic regression

## Abstract

**Background:**

Multimorbidity is the co-occurrence of two or more chronic diseases.

**Objective:**

This study, based on self-reported medical diagnosis, aims to investigate the dynamic distribution of multimorbidity across sociodemographic levels and its impacts on health-related issues over 15 years in Brazil using national data.

**Methods:**

Data were analyzed using descriptive statistics, hypothesis tests, and logistic regression. The study sample comprised 679,572 adults (18-59 years of age) and 115,699 elderly people (≥60 years of age) from the two latest cross-sectional, multiple-cohort, national-based studies: the National Sample Household Survey (PNAD) of 1998, 2003, and 2008, and the Brazilian National Health Survey (PNS) of 2013.

**Results:**

Overall, the risk of multimorbidity in adults was 1.7 times higher in women (odds ratio [OR] 1.73, 95% CI 1.67-1.79) and 1.3 times higher among people without education (OR 1.34, 95% CI 1.28-1.41). Multiple chronic diseases considerably increased with age in Brazil, and people between 50 and 59 years old were about 12 times more likely to have multimorbidity than adults between 18 and 29 years of age (OR 11.89, 95% CI 11.27-12.55). Seniors with multimorbidity had more than twice the likelihood of receiving health assistance in community services or clinics (OR 2.16, 95% CI 2.02-2.31) and of being hospitalized (OR 2.37, 95% CI 2.21-2.56). The subjective well-being of adults with multimorbidity was often worse than people without multiple chronic diseases (OR=12.85, 95% CI: 12.07-13.68). These patterns were similar across all 4 cohorts analyzed and were relatively stable over 15 years.

**Conclusions:**

Our study shows little variation in the prevalence of the multimorbidity of chronic diseases in Brazil over time, but there are differences in the prevalence of multimorbidity across different social groups. It is hoped that the analysis of multimorbidity from the two latest Brazil national surveys will support policy making on epidemic prevention and management.

## Introduction

It is estimated that around 70% of all deaths worldwide are caused by noncommunicable diseases (NCDs), mainly cardiovascular diseases (31%), cancers (16%), chronic respiratory diseases (7%), and diabetes (3%) [1]. In Brazil, cardiovascular diseases, cancers, chronic respiratory diseases, Alzheimer’s disease and other dementias, diabetes mellitus, chronic kidney disease, cirrhosis, and other chronic liver diseases represented 62.4% of all deaths between 1990 and 2017 [2].

The co-occurrence of two or more chronic diseases in an individual is called multimorbidity [3-5]. Cross-country comparisons are challenging, essentially because there is no standard definition of what diseases should be considered for multimorbidity [3]. According to a literature review [5], the most common diseases are diabetes, hypertension, variations of heart disease, hyperlipidemia, and obesity, there being a high variability of coexisting additional diseases. Individuals with multimorbidity could have a higher risk of polypharmacy and of having difficulty in managing special diets, and consequently, these factors could intensify the demand on health care resources and increase the vulnerability of patients to safety issues [6-8]. The association between multimorbidity and socioeconomic and demographic variables has been explored in the literature [9-11] using single-cohort data for the Brazilian population [12-17]. Brazil is the sixth-largest country in the world in territory and the fifth largest in terms of population [18]. However, there is little evidence on which to base the incidence of multimorbidity of chronic NCDs using multiple cohorts of data for Brazil.

The objective of this study is to evaluate dynamic distributions of the 9 key NCDs among the Brazilian population using integrated cross-sectional population surveys over a period of 15 years using the Brazilian National Household Sample Survey (PNAD) and the Brazilian National Health Survey (PNS). Our study developed statistical analysis to explore the possible nonclinical risk factors associated with the prevalence of multimorbidity. This is the first study to explore the temporal changes in multimorbidity over an extended period of 15 years, representing the largest sample (N=795,271) of multimorbidity research undertaken to date in Brazil. In addition, this study evaluated the impact of sociodemographic issues, well-being, health insurance, and health care demands on multimorbidity. A recent smaller-scale study of chronic diseases based on a single telephone survey corroborated our findings, considering a small scale of covariates [19].

## Methods

### Study Design and Data

PNAD and PNS are multiple-stage complex surveys conducted and made available online by the Brazilian Institute of Geography and Statistics to assess the status of households in Brazil. The National Research Ethics Commission approved the research and the content provided by all participants. The research used a stratified sampling method based on interviews conducted in all states and regions of Brazil. PNAD uses a 3-stage self-weighted cluster sampling technique. In the initial stage, cities with a large population and those belonging to metropolitan regions are included and other cities belonging to the same geographic microregion are grouped into a stratum of approximately the same size and systematically selected with probabilities proportional to their size. In the second stage, the census sectors are systematically chosen based on the last census in 2010, and the third sampling stage selects households in each sector [20]. PNS also uses a three-stage stratified sampling approach, which is a subsample of the Master Sample of the Integrated Household Surveys System consisting of primary sampling units (PSUs). In the first stage, PSU selection is obtained by simple random sampling. The second stage includes the selection of households. The last stage randomly selects a resident who is 18 years of age or above to answer the survey [21].

In this study, 4 cross-sectional national-based surveys from PNAD (1998, 2003, and 2008) and PNS (2013) were integrated for the common variables. To ensure the accuracy of analysis, variables that had over 25% missing values were excluded.

### Variables

NCD questions intend to measure current morbidity (self-declared), including back/column, arthritis/rheumatism, cancer, diabetes, bronchitis/asthma, high blood pressure (HBP, or hypertension), heart disease, chronic renal insufficiency (kidney failure), and depression. Multimorbidity means that the patient has two or more NCDs. Overall, 11 independent variables in each cohort (ie, gender, race/color, age group, region, literacy, employment, subjective well-being (SWB), health insurance, health service accessibility, health service need, and hospitalization) were considered for this study.

In the survey, literacy was used to assess educational background. Similarly, the study asked, “Have you had a job in the past 12 months?” to indicate participants’ employment status. To analyze the spatial feature of multimorbidity over time, we divided the nation into five regions, which are North, Northeast, Midwest, Southeast, and South, using the Brazilian government’s official rules on dividing the territory. Additionally, this study assessed the impacts of health service usage on multimorbidity. Understanding the distribution of multimorbidity among different groups can support health prevention policies to decrease multimorbidity and to save public expenditure on health services. We explored the recent usage of certain health services in the previous 2 weeks, the frequency of hospitalization in the past 12 months, and the frequency with which the same health center was visited. Most people in Brazil use public health services.

The ability to access private health care using health insurance represents the available financial resources for health management [22]. Consequently, this study was designed to use health insurance as an indicator of attitudes toward personal health management. A subject’s well-being was presented in the surveys using a 5-point scale (1 for the best to 5 for the worst) and was used to present the participants’ perception of their health. Some new variables were derived from the existing information in the database, based on the research objective (eg, group ages and group states in the official regions).

### Statistical Analysis

The analysis of multimorbidity was conducted from three aspects, which are social demographic, region, and utilization of health services. All analyses were categorized by age and gender. Descriptive analysis and data visualization were performed to identify differences in the participants’ behavior and to summarize each variable’s overlap distribution used in this study across each cohort. The sample comprised 795,271 participants divided into 2 main groups with 679,572 adults (18-59 years of age) and 115,699 elderly people (≥60 years of age). The inclusion criterion was being 18 years old or older, and we excluded 1.2% of individuals who did not answer all the questions about the 9 chronic diseases analyzed. The other 11 questions for analysis were mandatory in the surveys without any missing data. Hypothesis testing was conducted to determine the association between multimorbidity and demographic, geographic, socioeconomic, and health characteristics. The occurrences of all combined NCDs on each participant were explored using frequency analysis, and the interimpacts of NCDs were also tested. The Pearson chi-square test was used to determine the association between all variables and the occurrence of multimorbidity. Logistic regression models were applied for analyzing the impacts on multimorbidity. To estimate the association with multimorbidity, odds ratios (ORs) were computed with 95% CIs. First, the binary logistic regression model (BLRM) was used to compare the probability of multimorbidity occurrence (with: 1; without: 0) for the associated sociodemographic risk factors, such as gender, race/color, age group, region, literacy, employment, and health insurance. Second, the BLRM was applied to analyze the impacts of multimorbidity/sociodemographic characteristics on health-related factors, such as the participant’s well-being, health service accessibility, health service need, and hospitalization. This methodology enabled us to estimate the OR of each predictor variable, independent of all other variables in the model. *P* values (2-sided) with 95% CIs were used for statistical significance. The data were analyzed using SPSS Statistics and QGIS for map design.

## Results

### Descriptive Statistics of Multimorbidity

**Figure 1:**
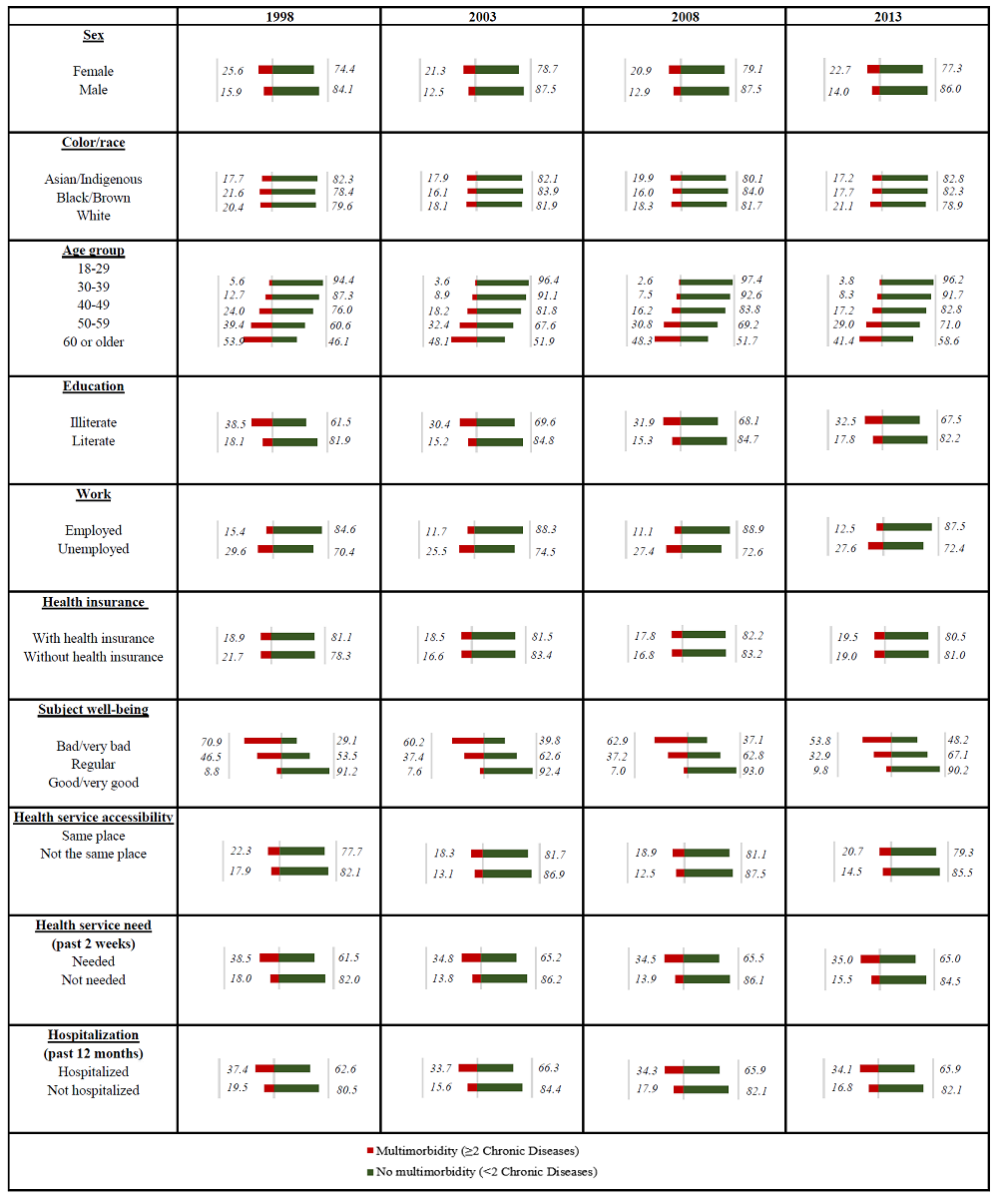
Percentage of multimorbidity by year with sociodemographics, subjective well-being, and health service characteristics.

Overall, 18.3% of the participants had 2 or more NCDs in Brazil, and the prevalence of multimorbidity varied between 17.1% and 21% over the 15 years of this study from 1998 to 2013 (see the multimorbidity frequency tables in Multimedia Appendix 1). The results show that 1 in every 5 Brazilian adults of 18 years and above had, on average, multimorbidity in 1998 and 2013, with a ratio of 1 in 6 in 2003 and 2008. Figure 1 summarizes the descriptive statistics of multimorbidity according to gender, race, age, education, and employment. In general, the percentage of multimorbidity for females varied between 20.9% and 25.6% and was higher than that of males over the study period. The difference in the multimorbidity rate between the genders varied between 9.7% (1998), 8.8% (2003), 8% (2008), and 8.7% (2013). The occurrence of multimorbidity grew gradually with age over the 15 years. However, the percentage of multimorbidity for young people increased in 2013 by 0.2% compared with that in 2008. The age group of 18-29 years had the lowest multimorbidity rate (2.6%) in 2008, and for people aged 60 or over, multimorbidity was lowest in 2013 (41.1%). The gap in the occurrence rate of having more than two NCDs between the lowest and highest age groups shrank from 48.3% in 1998 to 37.3% in 2013.

**Figure 2:**
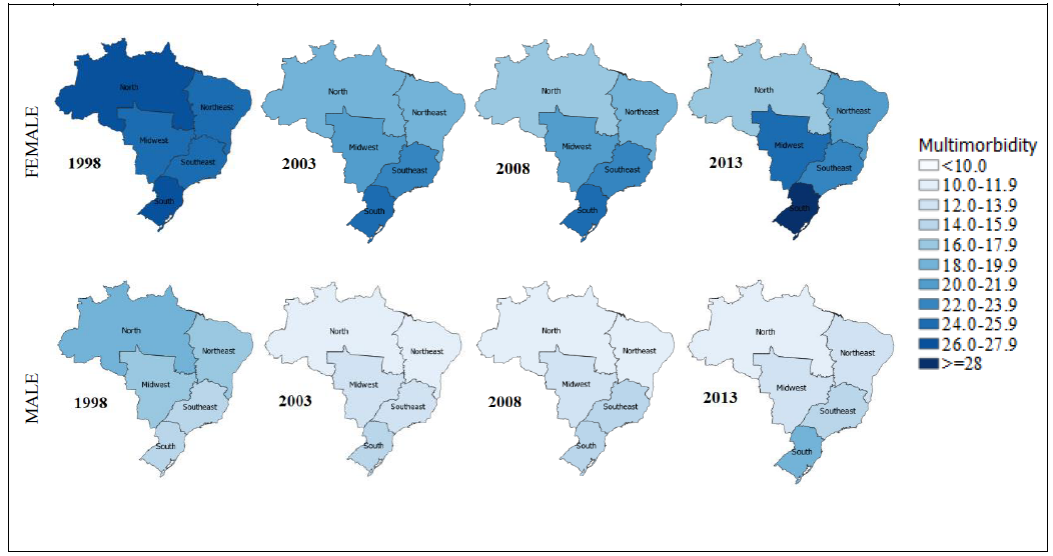
Percentage of multimorbidity in the 5 regions of Brazil by sex.

The least educated in the sample population (people without education) had a higher rate of multiple chronic diseases than the highest educated group in every study period (Figure 1). The greatest difference (20.4%) in the multimorbidity rate between the least and most educated groups occurred in 1998, and the rate gradually declined in subsequent years apart from 2008. The unemployed group had higher rates of multimorbidity in comparison with employed people for each individual period. The rate for both employed and unemployed people was the lowest in 2003 and increased slightly from 2003. With regard to the geographical distribution of multimorbidity, the South and Southeast had a higher rate of multimorbidity compared to the North and Northeast, except in 1998, and the pattern was the same across gender groups (Figure 2).

**Figure 3:**
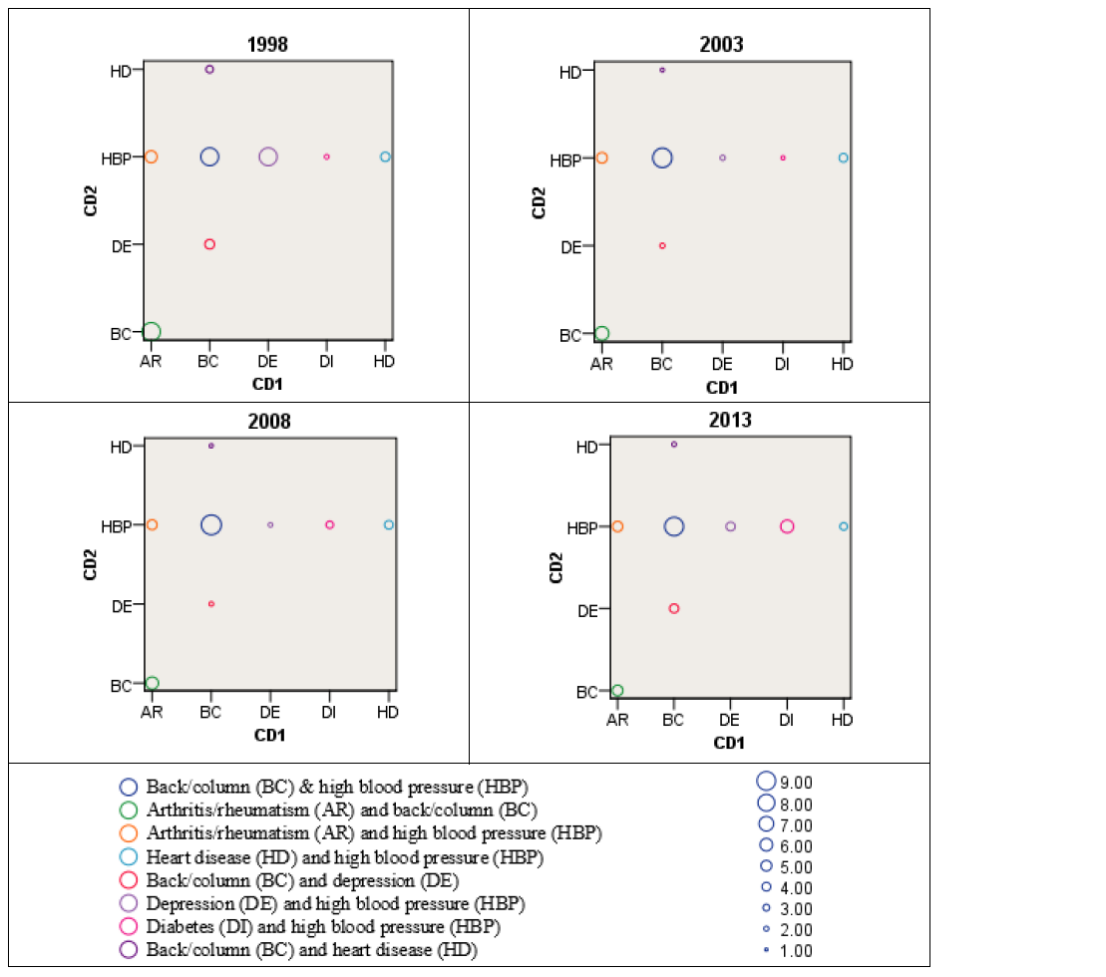
Most frequent CD combinations in Brazil. CD: chronic disease.

Pearson chi-square tests showed that multimorbidity was significantly associated with the risk factors considered over the 15-year period in Brazil (*P*<.001) except for health insurance in 2013. The covariates most associated with multimorbidity were age (0.415 in 2008) and SWB (0.483 in 1998). Clearly, participants with multiple NCDs considered their health status as bad or very bad more frequently than people without multimorbidity (Figure 1). The data also indicate that the demand for health services and hospitalization was higher among participants with multimorbidity. The minority of participants in the four cohorts declared they had visited health-related units, services, or professionals in the past 2 weeks or had been hospitalized in the past 12 months. Participants with back/column problems and HBP (7%) or column/back and arthritis/rheumatism (5.7%) were the most common of the multimorbidity population, as shown in Figure 3 (the frequency of each NCD calculated is shown in Multimedia Appendix 2). For multimorbidity with 3 chronic diseases, the most prevalent combination for the participants was arthritis/rheumatism, back/column diseases, and HBP (2.8%).

### Multimorbidity Valuation Model

We developed BLRMs to evaluate the impact of sociodemographics (risk factors) on multimorbidity in Brazil and their associations with health-related variables. The parameter estimations of BLRM analysis are explained in the following sections.

#### Sociodemographic Analysis

Tables 1 and 2 show the OR of multimorbidity over the period of 1998-2013 for adults and for the elderly, respectively. The odds of multimorbidity for female adults increased by 73% compared with males (OR 1.73). For those over 60 years, the chance of having multimorbidity was 52% higher for women than for men. The ORs of multimorbidity for Black/Brown adult Brazilians (OR 0.91) and the elderly (OR 0.72) were relevantly smaller. However, there was little difference in the occurrence of multimorbidity between the Asian/indigenous group and Whites in Brazil (adults: OR 1.09; elderly: OR 1.03). Overall, the prevalence of multimorbidity increased with age. For instance, the group aged between 50 and 59 years had an almost 12 times greater chance (OR 11.89) of developing multiple chronic diseases compared with people between 18 and 29 years old. However, the odds for those over 60 years were almost 12 times higher (OR 19.77) than for those between 18 and 29 years.

Regression analysis showed that illiteracy is positively associated with multimorbidity, and the odds of unemployed people having multimorbidity increased 1.5 times compared to the employed adults (OR 1.47). With regard to the distribution of multimorbidity in Brazil by region, people from areas in the South showed a greater chance (adults OR 1.35; elderly OR 1.53) of having multimorbidity than did people from the North, on average, over the whole period. Overall, participants with multiple chronic diseases were more likely to be uninsured (OR 1.12), except the elderly group (OR 0.93). Note that individuals of all ages in Brazil are entitled to public health insurance.

**Table 1:** Results of the binary logistic regression model examining the association with multimorbidity for Brazilian adults (18-59 years old).

Characteristics		1998			2003			2008			2013			Overall	
		OR_crude_^a^	OR_adjusted_^b^ (95% CI)	OR_crude_		OR_adjusted_ (95% CI)	OR_crude_		OR_adjusted_ (95% CI)	OR_crude_		OR_adjusted_ (95% CI)	OR_crude_		OR_adjusted_ (95% CI)
**Gender**															
	Male	1.00	1.00	1.00		1.00	1.00		1.00	1.00		1.00	1.00		1.00
	Female	1.83	1.77 (1.72-1.83)	1.97		1.82 (1.77-1.88)	1.79		1.60 (1.54-1.64)	1.88		1.79 (1.59-2.02)	1.86		1.73 (1.67-1.79)
	*P* value	—^c^	<.001	—		<.001	—		<.001	—		<.001	—		<.001
**Race/color**															
	White	1.00	1.00	1.00		1.00	1.00		1.00	1.00		1.00	1.00		1.00
	Black/Brown	0.71	0.66 (0.52-0.83)	1.11		0.99 (0.82-1.21)	1.16		1.12 (0.95-1.33)	0.89		0.94 (0.60-1.47)	0.97		0.91 (0.76-1.08)
	Asian/Indigenous	1.20	1.19 (1.14-1.23)	0.96		1.12 (1.08-1.16)	0.97		1.13 (1.09-1.67)	0.88		1.02 (0.91-1.15)	0.98		1.09 (1.05-1.13)
	*P* value	—	<.001	—		<.001	—		<.001	—		.89	—		<.001
**Age group (years)**															
	18-29	1.00	1.00	1.00		1.00	1.00		1.00	1.00		1.00	1.00		1.00
	30-39	2.54	2.66 (2.54-2.79)	2.71		2.80 (2.65-2.96)	2.98		3.10 (2.93-3.29)	2.32		2.41 (1.94-2.98)	2.61		2.70 (2.55-2.85)
	40-49	5.56	5.92 (5.65-6.20)	6.13		6.32 (6.00-6.65)	7.12		7.29 (6.91-7.70)	5.60		5.69 (4.64-6.99)	5.95		6.08 (5.77-6.40)
	50-59	11.37	11.61 (11.05-12.20)	13.29		13.14 (12.48-13.84)	16.42		15.86 (15.01-16.76)	10.23		10.13 (8.25-12.44)	12.18		11.89 (11.27-12.55)
	*P* value	—	<.001	—		<.001	—		<.001	—		<.001	—		<.001
**Education**															
	Literate	1.00	1.00	1.00		1.00	1.00		1.00	1.00		1.00	1.00		1.00
	Illiterate	2.30	1.39 (1.32-1.45)	1.84		1.20 (1.16-1.28)	1.94		1.23 (1.17-1.30)	1.83		1.13 (0.91-1.41)	2.02		1.34 (1.28-1.41)
	*P* value	—	<.001	—		<.001	—		<.001	—		.28	—		<.001
**Work**															
	Employed	1.00	1.00	1.00		1.00	1.00		1.00	1.00		1.00	1.00		1.00
	Unemployed	1.63	1.33 (1.29-1.38)	1.76		1.49 (1.44-1.54)	1.96		1.67 (1.61-1.73)	1.75		1.42 (1.26-1.60)	1.78		1.47 (1.42-1.52)
	*P* value	—	<.001	—		<.001	—		<.001	—		<.001	—		<.001
**Region**															
	North	1.00	1.00	1.00		1.00	1.00		1.00	1.00		1.00	1.00		1.00
	Northeast	0.84	0.70 (0.64-0.77)	0.90		0.78 (0.72-0.85)	1.04		0.94 (0.87-1.02)	1.09		0.98 (0.83-1.15)	1.01		0.90 (0.85-0.95)
	Southeast	0.66	0.59 (0.54-0.64)	1.07		0.91 (0.84-0.98)	1.30		1.16 (1.07-1.25)	1.32		1.20 (1.01-1.42)	1.10		0.99 (0.93-1.05)
	South	0.84	0.80 (0.72-0.88)	1.35		1.23 (1.13-1.33)	1.59		1.48 (1.35-1.62)	1.87		1.74 (1.43-2.12)	1.42		1.35 (1.26-1.44)
	Midwest	0.87	0.85 (0.77-0.94)	1.26		1.19 (1.10-1.30)	1.31		1.27 (1.16-1.39)	1.44		1.36 (1.14-1.62)	1.25		1.22 (1.15-1.30)
	*P* value	—	<.001	—		<.001	—		<.001	—		<.001	—		<.001
**Health insurance**															
	With insurance	1.00	1.00	1.00		1.00	1.00		1.00	1.00		1.00	1.00		1.00
	Without insurance	1.26	1.29(1.25-1.35)	0.95		1.05(1.01-1.09)	1.04		1.10(1.06-1.15)	1.05		1.07(0.95-1.20)	1.06		1.12(1.08-1.16)
	*P* value	—	<.001	—		.02	—		<.001	—		.27	—		<.001

^a^OR_crude_: crude odds ratio.

^b^OR_adjusted_: odds ratio adjusted for gender, color/race, age group, literacy, work, region and health insurance.

^c^Not applicable.

**Table 2:** Results of the binary logistic regression model examining the association with multimorbidity for Brazilian elderly (≥60 years old).

Characteristics		1998		2003		2008		2013		Overall	
		OR_crude_^a^	OR_adjusted_^b^ (95% CI)	OR_crude_	OR_adjusted_ (95% CI)	OR_crude_	OR_adjusted_ (95% CI)	OR_crude_	OR_adjusted_ (95% CI)	OR_crude_	OR_adjusted_ (95% CI)
**Gender**											
	Male	1.00	1.00	1.00	1.00	1.00	1.00	1.00	1.00	1.00	1.00
	Female	1.81	1.60 (1.52-1.68)	1.78	1.56 (1.49-1.64)	1.76	1.58 (1.51-1.65)	1.50	1.42 (1.22-1.65)	1.68	1.52 (1.44-1.60)
	*P* value	—^c^	<.001	—	<.001	—	<.001	—	<.001	—	<.001
**Race/color**											
	White	1.00	1.00	1.00	1.00	1.00	1.00			1.00	1.00
	Black/Brown	0.58	0.64 (0.47-0.85)	0.56	0.57 (0.43-0.75)	0.70	0.70 (0.57-0.87)	—	—	0.70	0.72 (0.57-0.91)
	Asian/Indigenous	1.18	1.10 (1.03-1.17)	0.94	1.05 (0.99-1.11)	0.93	1.06 (1.01-1.11)	—	—	0.95	1.03 (0.97-1.09)
	*P* value	—	<.001	—	<.001	—	<.001	—	—	—	<.001
**Education**											
	Literate	1.00	1.00	1.00	1.00	1.00	1.00	—	—	1.00	1.00
	Illiterate	1.49	1.32 (1.24-1.41)	1.11	1.15 (1.09-1.22)	1.05	1.12 (1.06-1.18)	—	—	1.18	1.23 (1.16-1.30)
	*P* value	—	<.001	—	<.001	—	<.001	—	—	—	<.001
**Work**											
	Employed	1.00	1.00	1.00	1.00	1.00	1.00	1.00	1.00	1.00	1.00
	Unemployed	1.79	1.55 (1.45-1.65)	1.98	1.70 (1.60-1.80)	1.90	1.66 (1.57-1.75)	1.54	1.42 (1.16-1.74)	1.76	1.53 (1.44-1.63)
	*P* value	—	<.001	—	<.001	—	<.001	—	.001	—	<.001
**Region**											
	North	1.00	1.00	1.00	1.00	1.00	1.00	1.00	1.00	1.00	1.00
	Northeast	0.88	0.84 (0.73-0.95)	0.83	0.82 (0.74-0.91)	0.87	0.83 (0.75-0.91)	1.38	1.34 (1.08-1.67)	1.06	1.01 (0.93-1.10)
	Southeast	0.74	0.78 (0.69-0.89)	1.01	1.00 (0.90-1.11)	1.09	1.04 (0.94-1.14)	1.78	1.75 (1.40-2.18)	1.22	1.21 (1.11-1.33)
	South	0.87	0.96 (0.84-1.11)	1.21	1.27 (1.13-1.42)	1.28	1.27 (1.14-1.41)	2.28	2.28 (1.77-2.92)	1.49	1.53 (1.39-1.69)
	Midwest	0.89	0.96 (0.82-1.11)	1.16	1.20 (1.06-1.35)	1.22	1.20 (1.07-1.34)	1.64	1.63 (1.28-2.07)	1.29	1.31 (1.19-1.44)
	*P* value	—	<.001	—	<.001	—	<.001	—	<.001	—	<.001
**Health insurance**											
	With insurance	1.00	1.00	1.00	1.00	1.00	1.00	1.00		1.00	1.00
	Without insurance	1.27	1.14 (1.07-1.22)	0.92	0.93 (0.88-0.99)	0.85	0.87 (0.82-0.92)	0.86	—	0.94	0.93 (0.88-0.99)
	*P* value	—	<.001	—	.02	—	<.001	—	—	—	.02

^a^OR_crude_: crude odds ratio.

^b^OR_adjusted_: odds ratio adjusted for gender, color/race, age group, literacy, work, region and health insurance.

^c^Not applicable.

#### SWB and Health Assistance

In general, the participants without multiple chronic diseases naturally considered their health status as either good or regular. However, the responses from the participants with multimorbidity showed that their SWB was almost 10 times more likely to be perceived as bad/very bad (adult OR 12.85; elderly OR 8.35) than good/very good in all study cohorts. Tables 3 and 4 shows the OR for adults and for the elderly, respectively. The odds of participants with multimorbidity using the same health care unit was about 30% greater than that of the other people. In addition, the participants with multimorbidity showed a 50% greater chance of needing health services (adult OR 2.73; elderly OR 2.16) and of being hospitalized (adult OR 2.29; elderly OR 2.37) compared with non-multimorbidity groups.

**Table 3:** Results of the binary logistic regression model examining the association with subjective well-being (SWB) and health service utilization for Brazilian adults (18-59 years old).

Status of variables			1998			2003			2008			2013			Overall	
		OR_crude_^a^		OR_adjusted_^b^ (95% CI)	OR_crude_		OR_adjusted_ (95% CI)	OR_crude_		OR_adjusted_ (95% CI)	OR_crude_		OR_adjusted_ (95% CI)	OR_crude_		OR_adjusted_ (95% CI)
**SWB (bad/very bad)**																
	Without multimorbidity	1.00		1.00	1.00		1.00	1.00		1.00	1.00		1.00	1.00		1.00
	With multimorbidity	21.32		13.90 (12.98-14.88)	17.29		12.81 (12.01-13.65)	24.04		18.17 (17.04-19.38)	12.08		10.02 (8.30-12.10)	17.70		12.85 (12.07-13.68)
	*P* value	—^c^		<.001	—		<.001	—		<.001	—		<.001	—		<.001
**Health service accessibility**																
	Without multimorbidity	1.00		1.00	1.00		1.00	1.00		1.00	1.00		1.00	1.00		1.00
	With multimorbidity	1.29		1.28 (1.23-1.33)	1.45		1.32 (1.26-1.38)	1.57		1.43 (1.38-1.49)	1.42		1.33 (1.17-1.52)	1.41		1.31 (1.26-1.36)
	*P* value	—		<.001	—		<.001	—		<.001	—		<.001	—		<.001
**Health service need**																
	Without multimorbidity	1.00		1.00	1.00		1.00	1.00		1.00	1.00		1.00	1.00		1.00
	With multimorbidity	2.90		2.67 (2.57-2.77)	3.35		2.82 (2.72-2.93)	3.42		2.98 (2.87-3.09)	3.06		2.62 (2.33-2.94)	3.16		2.73 (2.63-2.83)
	*P* value	—		<.001	—		<.001	—		<.001	—		<.001	—		<.001
**Hospitalization**																
	Without multimorbidity	1.00		1.00	1.00		1.00	1.00		1.00	1.00		1.00	1.00		1.00
	With multimorbidity	2.28		2.22 (2.11-2.33)	2.61		2.46 (2.35-2.58)	2.71		2.64 (2.52-2.77)	2.71		1.89 (1.61-2.22)	2.40		2.29 (2.19-2.39)
	*P* value	—		<.001	—		<.001	—		<.001	—		<.001	—		<.001

^a^OR_crude_: crude odds ratio.

^b^OR_adjusted_: odds ratio adjusted for gender, color/race, age group, literacy, work, region and health insurance.

^c^Not applicable.

**Table 4:** Results of the binary logistic regression model examining the association with subjective well-being (SWB) and health service utilization for Brazilian elderly (≥60 years old).

Status of variables			1998			2003			2008			2013			Overall	
		OR_crude_^a^		OR_adjusted_^b^ (95% CI)	OR_crude_		OR_adjusted_ (95% CI)	OR_crude_		OR_adjusted_ (95% CI)	OR_crude_		OR_adjusted_ (95% CI)	OR_crude_		OR_adjusted_ (95% CI)
**SWB (bad/very bad)**																
	Without multimorbidity	1.00		1.00	1.00		1.00	1.00		1.00	1.00		1.00	1.00		1.00
	With multimorbidity	11.66		11.4 9 (10.41-12.69)	7.61		8.81 (8.07-9.63)	7.60		9.16 (8.43-9.96)	4.95		6.23 (4.93-7.86)	7.24		8.35 (7.69-9.07)
	*P* value	—^c^		<.001	—		<.001	—		<.001	—		<.001	—		<.001
**Health service accessibility**																
	Without multimorbidity	1.00		1.00	1.00		1.00	1.00		1.00	1.00		1.00	1.00		1.00
	With multimorbidity	1.36		1.38 (1.30-1.48)	1.55		1.43 (1.34-1.52)	1.51		1.43 (1.35-1.51)	1.25		1.17 (0.98-1.40)	1.38		1.30 (1.23-1.38)
	*P* value	—		<.001	—		<.001	—		<.001	—		.08	—		<.001
**Health service need**																
	Without multimorbidity	1.00		1.00	1.00		1.00	1.00		1.00	1.00		1.00	1.00		1.00
	With multimorbidity	2.56		2.59 (2.41-2.78)	2.78		2.62 (2.47-2.79)	2.58		2.46 (2.32-2.60)	1.81		1.71 (1.44-2.02)	2.26		2.16 (2.02-2.31)
	*P* value	—		<.001	—		<.001	—		<.001	—		<.001	—		<.001
**Hospitalization**																
	Without multimorbidity	1.00		1.00	1.00		1.00	1.00		1.00	1.00		1.00	1.00		1.00
	With multimorbidity	2.32		2.32 (2.14-2.52)	2.55		2.47 (2.29-2.66)	2.46		2.39 (2.23-2.56)	2.32		2.31 (1.86-2.88)	2.43		2.37 (2.21-2.56)
	*P* value	—		<.001	—		<.001	—		<.001	—		<.001	—		<.001

^a^OR_crude_: crude odds ratio.

^b^OR_adjusted_: odds ratio adjusted for gender, color/race, age group, literacy, work, region and health insurance.

^c^Not applicable.

## Discussion

### Principal Findings

The main findings of this study were that in Brazil, multimorbidity was higher among women, people without education, and the unemployed. We confirmed that multiple chronic diseases grow considerably with age. Similarly, people with multimorbidity made greater use of health assistance in community services or clinics and of hospitalization. The people with multimorbidity often rated their SWB worse than people without multiple chronic diseases. These patterns were similar across all four cohorts analyzed. The literature indicates a higher prevalence of multimorbidity in groups of women, older people, and people with lower socioeconomic status in different countries [11,23,24], even though most studies are from Europe and North America.

The multimorbidity rate in Brazil gradually declined from 1998 to 2008, yet increased in 2013 by 2%, and was consistently characterized by gender, age, region, well-being, health service utilization, and health insurance across the 15-year period. Our study shows that the effect of multimorbidity has shifted little over the 15 years. Based on the expected population growth [20], we estimated that the multimorbidity rate would increase to 20.6% in 2020 and 21.9% in 2025, having considered the expected aging population (30.2 million in 2020 and 36.1 million in 2025), respectively. It is estimated that between 16% and 57% of adults in developed countries have multiple chronic conditions [25]. The Brazilian females had around 70% more chance of having multimorbidity than the males, and the ORs were considerably higher for women and grew with age in the period of 1998-2013.

We believe that differences in multimorbidity occurrences between gender and the higher use of health care services by individuals with multimorbidity will remain largely unchanged in the near future in Brazil, as the distribution between these groups is not expected to change [20]. Given the higher life expectancy for women (80.25 years in 2020 and 81.22 years in 2025) [20], multiple chronic diseases create additional expenses for the health care system [26]. In Switzerland, the total health care costs were 5.5 times higher for individuals with multimorbidity [27]. Participants who self-declared as Black/Brown presented less chance of multimorbidity in comparison to those who self-declared as White in this study. Race is not entirely a determinant factor worldwide [28], but it might reflect that social conditions and income are important factors associated with multimorbidity. Blacks/Browns in Brazil have lower income and less opportunity to access health insurance, and worse housing conditions [29]. This is the only study that has explored the association between multimorbidity and literacy in Brazil, and it is alarming that Brazilian adults without education have more chance of developing multimorbidity. These socioeconomic factors increase exposure to risks and may lead to a reduced awareness of health status and care.

A range of different combinations of diseases has been presented in the literature, including clusters with cardiovascular and metabolic diseases, mental health problems, and musculoskeletal disorders [5]. For the individual NCD, this study found that the two most common sources are arterial hypertension (HBP; 16.5%, 18.0%, 19.9%, and 23.9%) and diabetes (3.1%, 3.8%, 5.1%, and 7.2%) in the respective cohorts. However, the most common combination of three NCDs are (1) back/column with HBP, (2) arthritis/rheumatism with column/back, and (3) arthritis/rheumatism with HBP (4.3%). The VIGITEL survey [19] indicated that the occurrence of HBP and diabetes was 24.7% and 7.7%, respectively, using telephone survey data collected from 33,356 participants in 26 state capitals and federal districts in 2018. It confirms that our findings are also in line with the most recent statistics of morbidity in Brazil. Unemployment does increase the risk of multimorbidity and is comparable to another Brazilian study [13] in which the unemployed showed a greater prevalence of multimorbidity. Over the study period, the prevalence gradually increased in the South and declined in the North. The regional prevalence of multimorbidity was only statistically different in 2003 and 2008. We believe that it would be interesting to observe the culture, lifestyle, and socioeconomic factors in future studies to better understand the distribution variations by geographic region. Considering the economic factors across those regions, southern Brazil had greater economic development but its multimorbidity occurrences were also the highest in Brazil.

The analysis of health characteristics and behaviors has not been explored in previous multimorbidity Brazilian studies using data from PNAD and PNS. The SWB should reflect not only NCDs but also communicable diseases and other health problems included in this study. Yet, there was a significant relationship between the SWB and multimorbidity for all four cohorts. This is the first study to analyze the association between the prevalence of multimorbidity and the SWB in the Brazilian national population. Individuals with multimorbidity perceived their SWB to be much worse than that of other groups, even though no causal relationship could be established from cross-sectional surveys.

It is gratifying to note that people with multimorbidity could access a range of health care services via the insurance system, although it was unclear whether insurance was purchased before or after they had multiple NCDs. Globally, having a health plan can generate greater access to health care services to reduce the risk of health hazards and improve the quality of life [22]. According to the supplementary national health agency [30], only 47 million Brazilians have health insurance (22.6%) out of a total population of about 208 million inhabitants [20]. The lack of health insurance could be attributed to income, as a considerable proportion of the Brazilian population cannot afford health care plans, even people who have multiple chronic diseases. The number of individuals with voluntary private health insurance considerably varies between countries, such as 43% in Australia, 29% in Denmark, 86% in France, and 11% in the United Kingdom [22].

Brazilians with multimorbidity had about 30% more chance of using the same health care unit, three quarters of them used health services more frequently, and over half of them were hospitalized at least once. We believe these patterns will show up in big national survey data analysis over time and could support decision making on health management [31] by revealing the determinants of multimorbidity in terms of sociodemographic factors and health services. Taking a broader perspective, it is essential to consider the care of people with multimorbidity from the aspect of improving health management in primary care [6,32,33].

### Limitations

Although our study explored patterns in the distribution of multimorbidity over a 15-year period (1998-2013) in Brazil, it also had several limitations. The self-reporting of chronic diseases could introduce bias (eg, the participant may not be aware of the disease or may not have been previously diagnosed by a doctor, or there may be over reporting), even though self-reporting is widely used in public health research [34]. Another limitation is that some survey questions changed over the period, yet this modification is unlikely to impact the outcomes of the study (eg, “Has any doctor ever given you the diagnosis of…” to “Has any doctor or health professional said that you have…”). Moreover, modifications in the data collection methodology could influence the geographic evaluation and the calculation of prevalence of NCDs by region.

This research evaluated the impact of multimorbidity on various outcomes that could lead to possible reverse causality, and future studies are needed to address this issue. Our findings indicate a possible influence of social conditions on health awareness and, consequently, an effect on the self-reporting of multimorbidity. Consistent with the prior literature [7,35], our study shows that multimorbidity in Brazil has led to a higher use of health care systems. Multimorbidity relates to physical inactivity, tobacco/alcohol use, and unhealthy diets. The lifestyle of individuals and families also needs to be considered in policies to minimize the risk of chronic diseases [36]. Multimorbidity is a complex health care situation that will tend to deteriorate with the co-occurrence of chronic diseases. Multiple health conditions are more common in disadvantaged groups and contribute to health inequalities [6]. Due to the economic status, it is possible that Brazilians from the North are less aware of their health condition compared with those from the South, which reinforces the need for preventative actions [37] and better health care services. Future studies will benefit from the availability of new population-based studies of multimorbidity patterns in Brazil founded on this study. It will be of pressing concern to explore further the relationships between unemployment, income, and chronic diseases as they relate to the difficulties of finding work or maintaining employment over time.

### Conclusions

The distribution patterns of multimorbidity provide clear evidence of where there are differences in the prevalence of multimorbidity across different social groups. Our findings can help to shape existing public health policies to accommodate different preventative activities within health care services. This analysis also confirms that differences in the economic development model, including regional inequalities, education, and employment, have greatly damaged public health development in Brazil, a developing country. This study provides scientific evidence of preventative public health strategies in pandemic situations that are needed to significantly affect vulnerable groups with multimorbidity from both temporal and geographic perspectives.

## Appendix

Multimedia Appendix 1 Descriptive summary table: multimorbidity over time.

Multimedia Appendix 2 Chronic disease prevalence in Brazilian adults over time (>18 years old).

## Abbreviations

BLRM: binary logistic regression model
HBP: high blood pressure
NCD: noncommunicable disease
OR: odds ratio
PNAD: National Sample Household Survey
PNS: Brazilian National Health Survey
PSU: primary sampling unit
SWB: subjective well-being
