# Supplementary material for: Prevalence of Multimorbidity of Chronic Noncommunicable Diseases in Brazil: Population-Based Study
Source: JMIR Public Health Surveill. 2021 Nov 25;7(11):e29693. doi: 10.2196/29693 (PMC8663437; doi:10.2196/29693)
Supplement: Multimedia Appendix 1 [file publichealth_v7i11e29693_app1.docx]

**Multimedia Appendix 1.** Descriptive summary table: multimorbidity over time.

|  | | No multimorbidity: <2 CD^a^ | | | | | | Multimorbidity: ≥2 CD | | | | |
| --- | --- | --- | --- | --- | --- | --- | --- | --- | --- | --- | --- | --- |
| Characteristic | | 1998 | 2003 | 2008 | 2013 | Overall | 1998 | | 2003 | 2008 | 2013 | Overall |
| **Sex** | | | | | | | | | | | | |
|  | Female (n) | 84,708 | 105,025 | 112,720 | 23,499 | 325,952 | 29,080 | | 28,382 | 29,696 | 6903 | 94,061 |
|  | Male (n) | 87,161 | 106,158 | 112,423 | 18,222 | 323,964 | 16,487 | | 15,158 | 16,680 | 2969 | 51,294 |
|  | Female (%) | 74.4 | 78.7 | 79.1 | 77.3 | 77.6 | 25.6 | | 21.3 | 20.9 | 22.7 | 22.4 |
|  | Male (%) | 84.1 | 87.5 | 87.1 | 86.0 | 86.3 | 15.9 | | 12.5 | 12.9 | 14.0 | 13.7 |
| **Color/race** | | | | | | | | | | | | |
|  | Asian/Indigenous (n) | 1247 | 1347 | 1912 | 650 | 5156 | 268 | | 294 | 476 | 135 | 1173 |
|  | Black/Brown (n) | 78,838 | 106,613 | 120,796 | 24,031 | 330,278 | 21,744 | | 20,449 | 23,029 | 5167 | 70,389 |
|  | White (n) | 91,784 | 103,223 | 102,435 | 17,040 | 314,482 | 23,555 | | 22,797 | 22,871 | 4570 | 73,793 |
|  | Asian/Indigenous (%) | 82.3 | 82.1 | 80.1 | 82.8 | 81.5 | 17.7 | | 17.9 | 19.9 | 17.2 | 18.5 |
|  | Black/Brown (%) | 78.4 | 83.9 | 84.0 | 82.3 | 82.4 | 21.6 | | 16.1 | 16.0 | 17.7 | 17.6 |
|  | White (%) | 79.6 | 81.9 | 81.7 | 78.9 | 81.0 | 20.4 | | 18.1 | 18.3 | 21.1 | 19.0 |
| **Age group (years)** | | | | | | | | | | | | |
|  | 18-29 (n) | 68,360 | 81,539 | 80,471 | 10,481 | 240,851 | 4018 | | 3006 | 2172 | 410 | 9606 |
|  | 30-39 (n) | 44,945 | 52,212 | 54,024 | 10,943 | 162,124 | 6520 | | 5126 | 4363 | 996 | 17,005 |
|  | 40-49 (n) | 29,922 | 38,250 | 43,430 | 8301 | 119,903 | 9468 | | 8494 | 8374 | 1722 | 28,058 |
|  | 50-59 (n) | 15,318 | 21,007 | 25,909 | 5835 | 68,069 | 9970 | | 10,067 | 11,531 | 2,388 | 33,956 |
|  | 60 or older (n) | 13,324 | 18,175 | 21,309 | 6161 | 58,969 | 15,591 | | 16,847 | 19,936 | 4,356 | 56,730 |
|  | 18-29 (%) | 94.4 | 96.4 | 97.4 | 96.2 | 96.2 | 5.6 | | 3.6 | 2.6 | 3.8 | 3.8 |
|  | 30-39 (%) | 87.3 | 91.1 | 92.5 | 91.7 | 90.5 | 12.7 | | 8.9 | 7.5 | 8.3 | 9.5 |
|  | 40-49 (%) | 76.0 | 81.8 | 83.8 | 82.8 | 81.0 | 24.0 | | 18.2 | 16.2 | 17.2 | 19.0 |
|  | 50-59 (%) | 60.6 | 67.6 | 69.2 | 71.0 | 66.7 | 39.4 | | 32.4 | 30.8 | 29.0 | 33.3 |
|  | 60 or older (%) | 46.1 | 51.9 | 51.7 | 58.6 | 51.0 | 53.9 | | 48.1 | 48.3 | 41.4 | 49.0 |
| **Education (literate)** | | | | | | | | | | | | |
|  | Illiterate (n) | 18,991 | 22,008 | 19,540 | 3151 | 63,690 | 11,881 | | 9599 | 9137 | 1516 | 32,133 |
|  | Literate (n) | 152,878 | 189,175 | 205,603 | 38,570 | 586,226 | 33,686 | | 33,941 | 37,239 | 8,356 | 113,222 |
|  | Illiterate (%) | 61.5 | 69.6 | 68.1 | 67.5 | 66.5 | 38.5 | | 30.4 | 31.9 | 32.5 | 33.5 |
|  | Literate (%) | 81.9 | 84.8 | 84.7 | 82.2 | 83.8 | 18.1 | | 15.2 | 15.3 | 17.8 | 16.2 |
| **Work** | | | | | | | | | | | | |
|  | Employed (n) | 111,826 | 137,278 | 153,164 | 25,245 | 427,513 | 20,301 | | 18,194 | 19,149 | 3604 | 61,248 |
|  | Unemployed (n) | 60,043 | 73,905 | 71,979 | 16,476 | 222,403 | 25,266 | | 25,346 | 27,227 | 6268 | 84,107 |
|  | Employed (%) | 84.6 | 88.3 | 88.9 | 87.5 | 87.5 | 15.4 | | 11.7 | 11.1 | 12.5 | 12.5 |
|  | Unemployed (%) | 70.4 | 74.5 | 72.6 | 72.4 | 72.6 | 29.6 | | 25.5 | 27.4 | 27.6 | 27.4 |
| **Health insurance** | | | | | | | | | | | | |
|  | With insurance (n) | 46,956 | 53,940 | 60,527 | 12,445 | 173,868 | 10,917 | | 12,251 | 13,066 | 3023 | 39,257 |
|  | Without insurance (n) | 124,913 | 157,243 | 164,616 | 29,276 | 476,048 | 34,650 | | 31,289 | 33,310 | 6849 | 106,098 |
|  | With insurance (%) | 81.1 | 81.5 | 82.2 | 80.5 | 81.6 | 18.9 | | 18.5 | 17.8 | 19.5 | 18.4 |
|  | Without insurance (%) | 78.3 | 83.4 | 83.2 | 81.0 | 81.8 | 21.7 | | 16.6 | 16.8 | 19.0 | 18.2 |
| **SWB^b^** | | | | | | | | | | | | |
|  | Bad/very bad (n) | 3388 | 4821 | 5104 | 1564 | 14,877 | 8242 | | 7286 | 8655 | 1823 | 26,006 |
|  | Regular (n) | 27,111 | 37,323 | 40,741 | 9696 | 114,871 | 23,604 | | 22,256 | 24,159 | 4753 | 74,772 |
|  | Good/very good (n) | 141,370 | 169,039 | 179,298 | 30,461 | 520,168 | 13,721 | | 13,998 | 13,562 | 3296 | 44,577 |
|  | Bad/very bad (%) | 29.1 | 39.8 | 37.1 | 46.2 | 36.4 | 70.9 | | 60.2 | 62.9 | 53.8 | 63.6 |
|  | Regular (%) | 53.5 | 62.6 | 62.8 | 67.1 | 60.6 | 46.5 | | 37.4 | 37.2 | 32.9 | 39.4 |
|  | Good/very good (%) | 91.2 | 92.4 | 93.0 | 90.2 | 92.1 | 8.8 | | 7.6 | 7.0 | 9.8 | 7.9 |
| **Health service accessibility** | | | | | | | | | | | | |
|  | Same place (n) | 177,865 | 160,767 | 158,197 | 30,727 | 467,556 | 33,826 | | 35,920 | 36,775 | 8004 | 114,525 |
|  | Not the same place (n) | 54,004 | 50,416 | 66,946 | 10,994 | 182,360 | 11,741 | | 7620 | 9601 | 1868 | 30,830 |
|  | Same place (%) | 77.7 | 81.7 | 81.1 | 79.3 | 80.3 | 22.3 | | 18.3 | 18.9 | 20.7 | 19.7 |
|  | Not the same place (%) | 82.1 | 86.9 | 87.5 | 85.5 | 85.5 | 17.9 | | 13.1 | 12.5 | 14.5 | 14.5 |
| **Health service need (past 2 weeks)** | | | | | | | | | | | | |
|  | Needed (n) | 19,467 | 26,009 | 27,458 | 6211 | 79,145 | 12,168 | | 13,865 | 14,467 | 3338 | 43,838 |
|  | Not needed (n) | 152,402 | 185,174 | 197,685 | 35,510 | 570,771 | 33,399 | | 29,675 | 31,909 | 6534 | 101,517 |
|  | Needed (%) | 61.5 | 65.2 | 65.5 | 65.0 | 64.4 | 38.5 | | 34.8 | 34.5 | 35.0 | 35.6 |
|  | Not needed (%) | 82.0 | 86.2 | 86.1 | 84.5 | 84.9 | 18.0 | | 13.8 | 13.9 | 15.5 | 15.1 |
| **Hospitalization (past 12 months)** | | | | | | | | | | | | |
|  | Hospitalized (n) | 11,444 | 13,631 | 14,170 | 2589 | 41,834 | 6826 | | 6926 | 7394 | 1340 | 22,486 |
|  | Not hospitalized (n) | 160,425 | 197,552 | 210,973 | 39,132 | 608,082 | 38,741 | | 36,614 | 38,982 | 8,532 | 122,869 |
|  | Hospitalized (%) | 62.6 | 66.3 | 65.7 | 65.9 | 65.0 | 37.4 | | 33.7 | 34.3 | 34.1 | 35.0 |
|  | Not hospitalized (%) | 80.5 | 84.4 | 84.4 | 82.1 | 83.2 | 19.5 | | 15.6 | 17.9 | 16.8 | 16.8 |
|  | *Total (n)* | *171,869* | *211,183* | *225,143* | *41,721* | *649,916* | *45,567* | | *43,540* | *46,376* | *9872* | *145,355* |
|  | *% Total* | *79.0* | *82.9* | *82.9* | *80.9* | *81.7* | *21.0* | | *17.1* | *17.1* | *19.1* | *18.3* |

^a^CD: chronic disease.

^b^SWB: subjective well-being.
