# Supplementary material for: Prevalence of Multimorbidity of Chronic Noncommunicable Diseases in Brazil: Population-Based Study
Source: JMIR Public Health Surveill. 2021 Nov 25;7(11):e29693. doi: 10.2196/29693 (PMC8663437; doi:10.2196/29693)
Supplement: Multimedia Appendix 2 [file publichealth_v7i11e29693_app2.docx]

**Multimedia Appendix 2.** CD^a^ prevalence in Brazilian adults over time (>18 years old).

| CD | | 1998 | 2003 | 2008 | 2013 | Overall |
| --- | --- | --- | --- | --- | --- | --- |
| **Arterial hypertension** | | | | | | |
|  | No (n) | 181,559 | 209,71 | 218,873 | 39,633 | 649,976 |
|  | Yes (n) | 35,837 | 44,852 | 52,646 | 11,960 | 145,295 |
|  | No (%) | 83.5 | 82.4 | 80.6 | 76.8 | 81.7 |
|  | Yes (%) | 16.5 | 17.6 | 19.4 | 23.2 | 18.3 |
| **Arthritis/rheumatism** | | | | | | |
|  | No (n) | 190,098 | 231,956 | 249,915 | 47,852 | 719,821 |
|  | Yes (n) | 27,338 | 22,767 | 21,604 | 3741 | 75,450 |
|  | No (%) | 87.4 | 91.1 | 92.0 | 92.7 | 90.5 |
|  | Yes (%) | 12.6 | 8.9 | 8.0 | 7.3 | 9.5 |
| **Asthma/bronchitis** | | | | | | |
|  | No (n) | 208,490 | 244,300 | 260,933 | 49,310 | 763,033 |
|  | Yes (n) | 8946 | 10,423 | 10,586 | 2283 | 32,238 |
|  | No (%) | 95.9 | 95.9 | 96.1 | 95.6 | 95.9 |
|  | Yes (%) | 4.1 | 4.1 | 3.9 | 4.4 | 4.1 |
| **Back/spine** | | | | | | |
|  | No (n) | 159,893 | 205,752 | 221,370 | 42,288 | 629,303 |
|  | Yes (n) | 57,543 | 48,971 | 50,149 | 9305 | 165,968 |
|  | No (%) | 73.5 | 80.8 | 81.5 | 82.0 | 79.1 |
|  | Yes (%) | 26.5 | 19.2 | 18.5 | 18.0 | 20.9 |
| **Cancer** | | | | | | |
|  | No (n) | 216,748 | 253,232 | 269,461 | 50,612 | 790,053 |
|  | Yes (n) | 688 | 1491 | 2058 | 981 | 5218 |
|  | No (%) | 99.7 | 99.4 | 99.2 | 98.1 | 99.3 |
|  | Yes (%) | 0.3 | 0.6 | 0.8 | 1.9 | 0.7 |
| **Chronic renal failure** | | | | | | |
|  | No (n) | 209,217 | 248,643 | 266,997 | 50,826 | 775,683 |
|  | Yes (n) | 8219 | 6080 | 4522 | 767 | 19,588 |
|  | No (%) | 96.2 | 97.6 | 98.3 | 98.5 | 97.5 |
|  | Yes (%) | 3.8 | 2.4 | 1.7 | 1.5 | 2.5 |
| **Depression** | | | | | | |
|  | No (n) | 200,702 | 239,922 | 256,605 | 47,664 | 744,893 |
|  | Yes (n) | 16,734 | 14,801 | 14,914 | 3929 | 50,378 |
|  | No (%) | 92.3 | 94.2 | 94.5 | 92.4 | 93.7 |
|  | Yes (%) | 7.7 | 5.8 | 5.5 | 7.6 | 6.3 |
| **Diabetes** | | | | | | |
|  | No (n) | 210,925 | 245,328 | 258,433 | 47,985 | 762,671 |
|  | Yes (n) | 6,511 | 9,395 | 13,086 | 3,608 | 32,600 |
|  | No (%) | 97.0 | 96.3 | 95.2 | 93.0 | 95.9 |
|  | Yes (%) | 3.0 | 3.7 | 4.8 | 7.0 | 4.1 |
| **Heart disease** | | | | | | |
|  | No (n) | 204,676 | 241,615 | 257,265 | 49,462 | 753,018 |
|  | Yes (n) | 12,760 | 13,108 | 14,254 | 2,131 | 42,253 |
|  | No (%) | 94.1 | 94.9 | 94.8 | 95.9 | 94.7 |
|  | Yes (%) | 5.9 | 5.1 | 5.2 | 4.1 | 5.3 |

^a^CD: chronic disease.
